# Rhodomycin A, a novel Src-targeted compound, can suppress lung cancer cell progression via modulating Src-related pathways

**DOI:** 10.18632/oncotarget.4761

**Published:** 2015-07-03

**Authors:** Yi-Hua Lai, Min-Hsuan Chen, Sih-Yin Lin, Sheng-Yi Lin, Yung-Hao Wong, Sung-Liang Yu, Huei-Wen Chen, Chih-Hsin Yang, Gee-Chen Chang, Jeremy J.W. Chen

**Affiliations:** ^1^ Institute of Biomedical Sciences, National Chung Hsing University, Taichung, Taiwan; ^2^ Division of Chest Medicine, Department of Internal Medicine, Taichung Veterans General Hospital, Taichung, Taiwan; ^3^ Agricultural Biotechnology Center, National Chung Hsing University, Taichung, Taiwan; ^4^ Department of Clinical and Laboratory Sciences and Medical Biotechnology, National Taiwan University College of Medicine, Taipei, Taiwan; ^5^ Graduate Institute of Toxicology, National Taiwan University College of Medicine, Taipei, Taiwan; ^6^ Department of Internal Medicine, National Taiwan University Hospital, Taipei, Taiwan; ^7^ Faculty of Medicine, School of Medicine, National Yang-Ming University, Taipei, Taiwan; ^8^ Rong Hsing Research Center for Translational Medicine, National Chung Hsing University, Taichung, Taiwan

**Keywords:** Src, EGFR, lung cancer, rhodomycin A, gefitinib

## Abstract

Src activation is involved in cancer progression and the interplay with EGFR. Inhibition of Src activity also represses the signalling pathways regulated by EGFR. Therefore, Src has been considered a target molecule for drug development. This study aimed to identify the compounds that target Src to suppress lung cancer tumourigenesis and metastasis and investigate their underlying molecular mechanisms. Using a molecular docking approach and the National Cancer Institute (NCI) compound dataset, eight candidate compounds were selected, and we evaluated their efficacy. Among them, rhodomycin A was the most efficient at reducing the activity and expression of Src in a dose-dependent manner, which was also the case for Src-associated proteins, including EGFR, STAT3, and FAK. Furthermore, rhodomycin A significantly suppressed cancer cell proliferation, migration, invasion, and clonogenicity *in vitro* and tumour growth *in vivo*. In addition, rhodomycin A rendered gefitinib-resistant lung adenocarcinoma cells more sensitive to gefitinib treatment, implying a synergistic effect of the combination therapy. Our data also reveal that the inhibitory effect of rhodomycin A on lung cancer progression may act through suppressing the Src-related multiple signalling pathways, including PI3K, JNK, Paxillin, and p130cas. These findings will assist the development of anti-tumour drugs to treat lung cancer.

## INTRODUCTION

Lung cancer is a predominant type of cancer that causes high mortality, and the survival rate remains relatively low even after surgery, chemotherapy or radiotherapy [1]. This low survival may result from the metastasis of cancer cells and arising resistance to drugs. Such obstacles make it difficult to effectively treat lung cancers. Recently, targeted therapy has shed some light on lung cancer treatment because of the close association between the occurrence and activation of oncogenes or the inhibition of tumour suppressors [2]. For example, epidermal growth factor receptor (EGFR) mutations or EGFR overexpression can be detected in non-small-cell lung cancer (NSCLC) patients, leading to the aberrant growth, metastasis, and resistance development of cancer cells [3]. Most of these mutations constitutively activate the kinase responsible for cellular signal transduction, demolishing cellular control over regulation. Therefore, small molecules against particular mutated kinase may be useful for improving lung cancer treatment [4].

Amongst the currently accessible drugs against lung cancer, geﬁtinib (Iressa) and erlotinib (Tarceva), so called EGF receptor tyrosine kinase inhibitors (EGFR-TKIs), are effective in patients with EGFR mutations, including exon 19 deletion and exon 21 substitution (L858R) [5]. EGFR-TKIs thwart the capacity of self-phosphorylation in EGFR, and in tandem, affect the downstream signalling cascade [6]. However, drug resistance appears to be inevitable. Studies have shown that EGFR with a T790M mutation, a secondary mutation in the EGFR kinase domain, is highly correlated with lung cancer relapse [7]. Although a drug mixture regimen improves the effectiveness of lung cancer treatment, more efforts are still needed to discover novel drugs or compounds that improve targeted therapy [8].

Src, a tyrosin kinase, is associated with the cellular growth, migration, and angiogenesis of tumour cells, making Src a potential target for lung cancer treatment [9]. Clinically, Src activation is commonly detected in NSCLC [10]. Additionally, once the expression level of Src is increased, a poor prognosis is observed in patients with NSCLC [11], colorectal [12] and breast cancers [13]. Due to the interactions between Src and EGFR, NSCLC treatment can be improved by suppressing Src [14]. This approach is strongly supported by a study in which apoptosis could be induced by adding a Src suppressor to disrupt the EGFR pathway [15]. Consequently, many candidates, such as dasatinib, saracatinib, bosutinib, KX2-391, XL999, XL288, and M475271, have been developed and subjected to clinical trials [16].

Src has been shown to be a potential target against lung cancer in light of its impacts on tumour growth by disrupting essential pathways. In this study, we first identified rhodomycin A by a molecular docking strategy and then investigated the functional roles of this drug in suppressing lung cancer progression and elucidated its molecular mechanisms. These results not only suggest potential new drugs but also unravel mechanisms to improve cancer treatment.

## RESULTS

### Virtual screening of potential candidate compounds

The chemical structures of NCI compound sets containing 46,827 antitumour drugs (http://www.dtp.nci.nih.gov/docs/cancer/cancer_data.html) were docked into the Src ATP binding site by the LibDock protocol of Discovery Studio v3.5. The derived LibDock score and consensus score were calculated based on the docking poses. Two ligands, dasatinib and imatinib, were adopted as the control ligands, in which candidate compounds must have a higher LibDock score and consensus score than the control ligands (Supplementary Table S1). Finally, we chose the top 8 compounds predicted to have the best performance in the virtual screening as candidate compounds, labelled N1 to N8. These candidate compounds were then utilised in the following biological assays for further screening.

**Figure 1:**
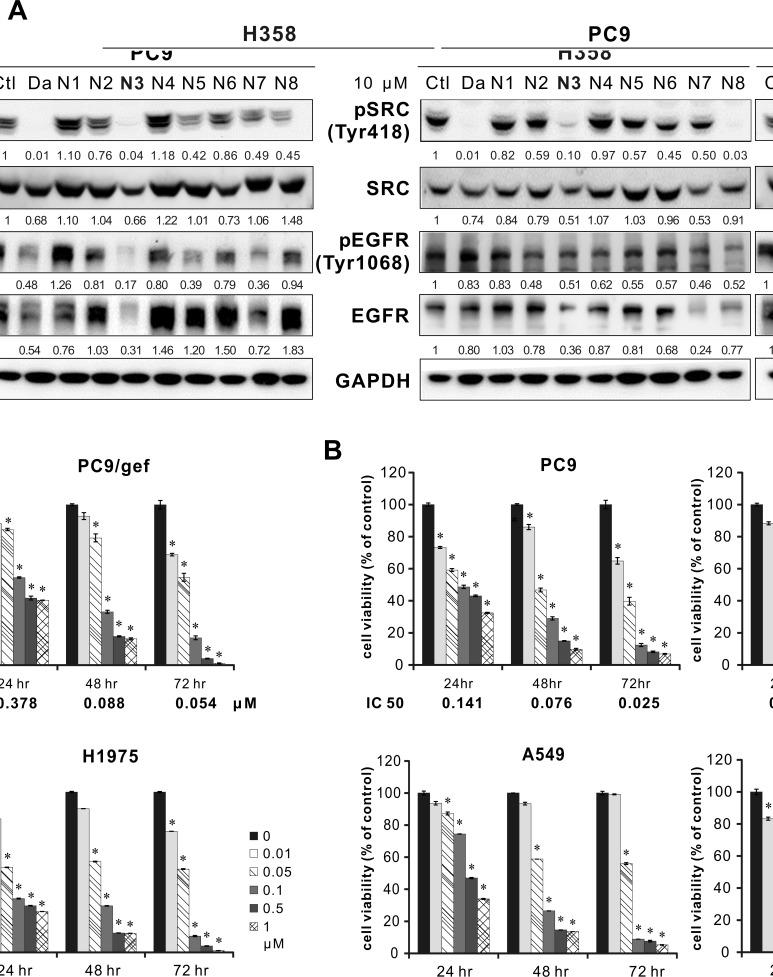
Inhibition of Src, EGFR, and cell viability by candidate compounds in different cell lines **A.** Western blotting of Src and EGFR in H358 and PC9 cells at 24 hours after candidate compound treatment. Ctl: 0.1% DMSO; Da: dasatinib, a positive control; and GAPDH: an internal control. **B.** Cell viability assay in NSCLC cell lines with varying EGFR status or drug resistance. The results are shown as the percentages of the control response (0 nM). The IC_50_ at 72 hours for PC9, PC9/gef, A549, and H1975 cells was 25, 22, 66, and 34 nM, respectively. Each treatment was independently performed in triplicate. *P < 0.05 compared with the control (0 nM, 0.1% DMSO).

The Western blot analyses showed that both N3 and N8 significantly decreased the phosphorylation of Src. Furthermore, N3 exhibited better performance in inhibiting EGFR and phosphor-EGFR than N8 in the two cell lines initially tested (Figure 1A). Therefore, we chose compound N3, i.e., rhodomycin A, to investigate its molecular mechanisms in Src activation and its effects on the related pathways essential for the growth and migration of tumour cells.

### Cytotoxic effect of rhodomycin A on cancer and noncancerous cells

To determine the proper concentration prior to the following experiments, we analysed the viability of PC9 (EGFR^exon19 del^; gefitinib-sensitive), PC9/gef (EGFR^exon19 del^; gefitinib-resistant), A549 (EGFR^wild-type^; gefitinib-resistant), and H1975 (EGFR^L858R+T790M^; gefitinib-resistant) lung adenocarcinoma cells for 24, 48, and 72 hours after exposure to rhodomycin A. Our results indicated that the cancer cell survival rate decreases in a dose-dependent manner. Notably, at 72 hours, 0.01 μΜ rhodomycin A resulted in a death rate of 20-30% for PC9, PC9/gef, and H1975 cells; 0.05 μΜ a death rate of > 40%; and > 0.1 μΜ a death rate of > 80%. The IC_50_ at each time point is shown in Figure 1B, and it is approximately tens to hundreds on a nM scale. For the non-tumoural BEAS2B cells, the IC_50_ was 1.02 μΜ at 24 hours, 0.1 μΜ at 48 hours, and 0.073 μΜ at 72 hours. Taken together, these experiments showed that rhodomycin A is less toxic for non-cancerous cells (Supplementary Figure S1).

### Rhodomycin A suppresses Src activity and alters the expression level of downstream proteins

**Figure 2:**
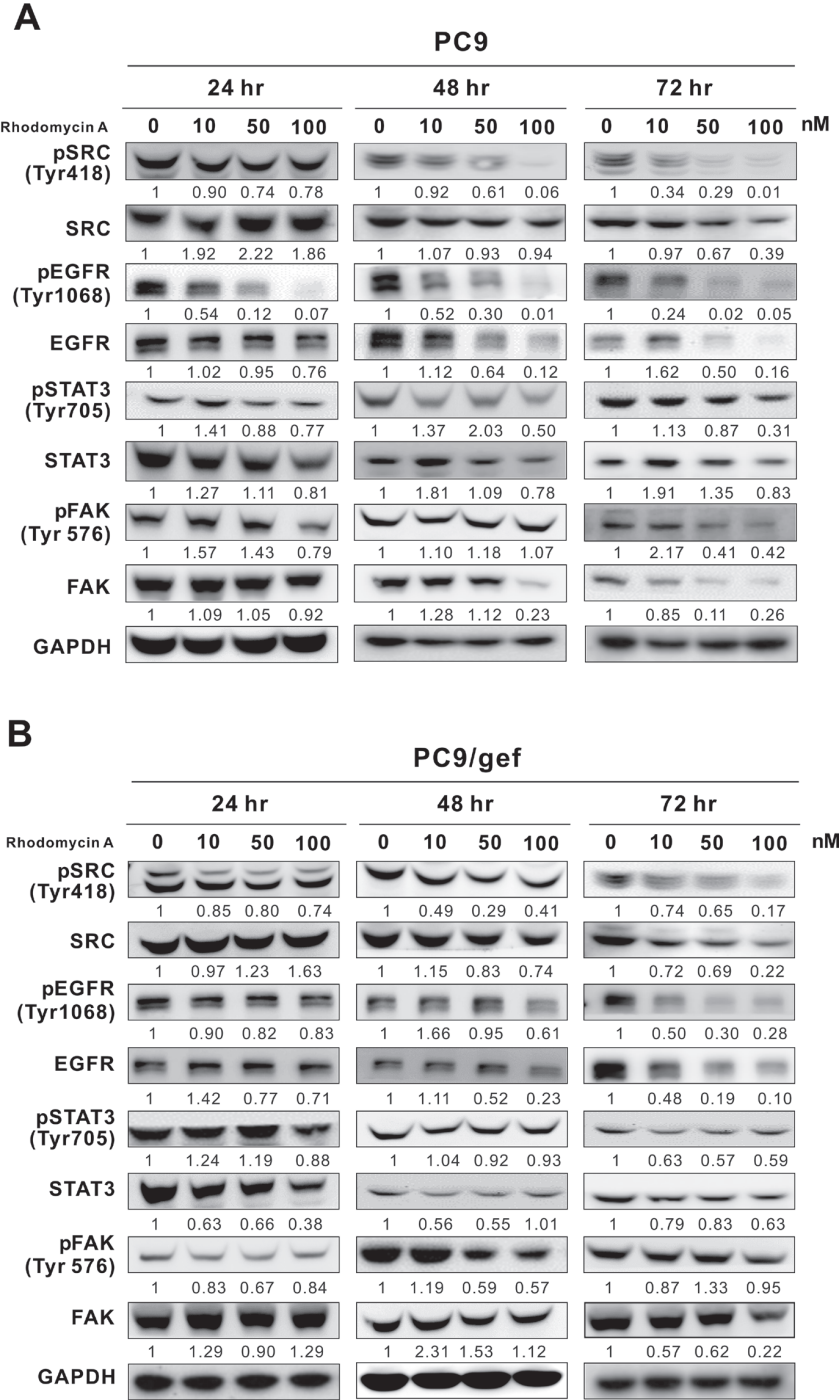
Effects of rhodomycin A on Src and its associated proteins The cells were treated with various concentrations of rhodomycin A for 24, 48, and 72 hours and subjected to Western blot analyses to determine the phosphorylation and expression levels of Src, EGFR, STAT3, and FAK in PC9 **A.** and PC9/gef cell lines **B.**. GAPDH was used as an internal control. Each treatment was independently performed in triplicate (0 nM represents 0.1% DMSO).

After the previous experiments, we used 10, 50, and 100 nM rhodomycin A to treat PC9 and PC9/gef for 24-72 hours, respectively. The Western blotting results showed that with increased compound concentrations and treatment time, the expression levels of pSrc, pEGFR, EGFR, pFAK, and FAK significantly decreased, whereas those of Src, pSTAT3, and STAT3 slightly decreased in PC9 cells (Figure 2A).

In the PC9/gef cell line, the gefitinib-resistant counterpart of PC9 cells, we discovered that the inhibitory pattern is somewhat different from the PC9 cells. The expression levels of p-Src, Src, p-EGFR, and EGFR were significantly reduced with the increase in the compound concentration and treatment time; nevertheless, those of p-STAT3, STAT3, p-FAK, and FAK were inhibited slightly (Figure 2B). Additionally, rhodomycin A slightly decreased the phosphorylation of Src, EGFR, STAT3, and FAK in dose-dependent manner in A549 cells and somewhat decreased the total level of expressed proteins, which was dependent on the time period of treatment (Supplementary Figure S2).

### Rhodomycin A suppresses the proliferation, invasion, and migration of cancer cells

**Figure 3:**
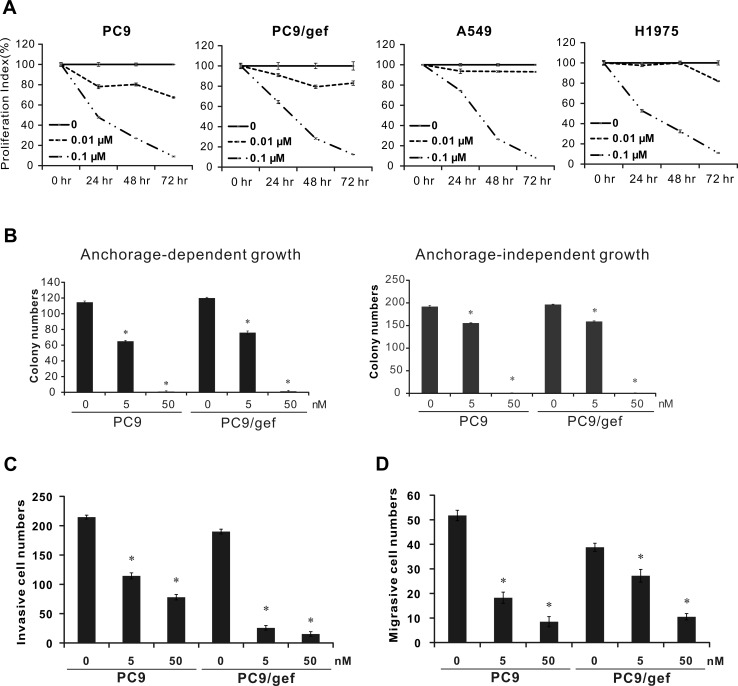
Inhibition of cancer cell proliferation, colony formation, invasion, and migration ability by rhodomycin A **A.** Proliferation assays. The proliferative abilities of PC9, PC9/gef, A549, and H1975 cells were examined with a PrestoBlue^®^ cell viability assay at 24, 48, or 72 hours. **B.** Colony formation in PC9 and PC9/gef cells. The cells grown in a culture dish with or without soft agar were treated with rhodomycin A and evaluated in clonogenic assays. The colonies with diameters ≥0.3 mm (anchorage-dependent) or ≥0.5 mm (anchorage-independent) were counted. **C.** Effect of rhodomycin A on cancer cell invasion as determined by a matrigel-coated transwell assay. **D.** Effect of rhodomycin A on cancer cell migration assessed on a non-coated transwell assay. Each treatment was independently performed in triplicate; 0 nM represents 0.1% DMSO. *P < 0.05 compared with vehicle-treated control (0 nM, 0.1% DMSO).

To investigate the anti-cancer effect of rhodomycin A, cell proliferation, colony formation, invasion, and migration analyses were performed. Rhodomycin A suppressed the proliferation of PC9, PC9/gef, A549, and H1975 cancer cells (Figure 3A). Moreover, it also inhibited the abilities of anchorage-dependent and -independent cell colony growth, regardless of whether the cells were gefitinib-sensitive (PC9) or -resistant (PC9/gef), even at a low concentration (Figure 3B). Similar results were observed in the A549 and CL1-5 lung cancer cells (Supplementary Figure S3, S4).

A previous study demonstrated that the activation and expression of Src could promotes cancer cell migration and invasion [17]. To determine the impacts of rhodomycin A on the migration and invasion of cancer cells, we treated PC9 and PC9/gef cells with various concentrations of rhodomycin A, which showed significant repression of cancer cell motility and invasiveness relative to control (Figure 3C and 3D).

### Rhodomycin A thwarts tumour growth and has a synergistic effect

**Figure 4:**
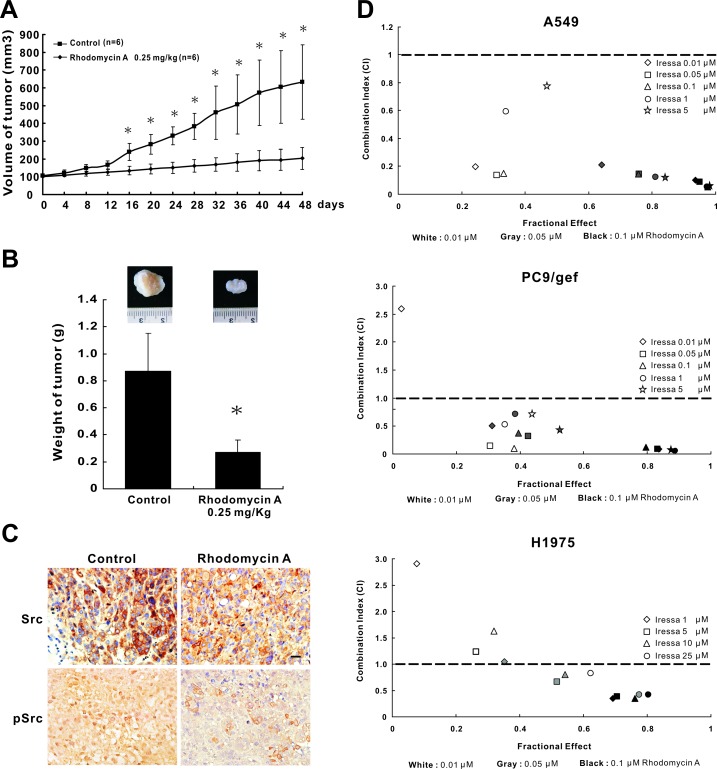
The effects of rhodomycin A on antitumour growth and synergism **A.** Tumourigenesis assay. Mice injected subcutaneously with 4 × 10^6^ live PC9/gef cells were divided into the DMSO-treated and drug-treated groups. The tumour volumes were measured every 4 days. Control: 0.1% DMSO, *n* = 6; rhodomycin A: 0.25 mg/kg, *n* = 6. *P < 0.05 compared with the vehicle-treated control. **B.** Tumour weight. The tumour weights are presented as the mean ± standard deviation. **C.** Immunohistochemistry of Src and p-Src showing staining in the cytoplasm, membrane and perinuclear region of paraffin-embedded murine tumour tissues under a light microscope (×400 magnification). The scale bars represent 100 μm. Control indicates 0.1% DMSO. **D.** The synergistic effect of rhodomycin A and gefitinib was determined by a PrestoBlue^®^ cell viability assay. Rhodomycin A (0.01, 0.05, and 0.1 μM), in combination with varying concentrations of gefitinib, was employed to treat gefitinib-resistant lung adenocarcinoma cell lines (A549, PC9/gef and H1975) for 72 hours; the cells were then evaluated by a proliferation assay. The data were further used to calculate the combination index (CI) using CalcuSyn software. Each treatment was independently performed in triplicate.

To examine the influence of rhodomycin A on tumour growth *in vivo*, PC9/gef cells were injected into SCID mice. The tumour volumes were measured every four days. The mice were randomly grouped into rhodomycin A-treated (p.o., 0.25 mg/kg/day) or control groups. The mean size and weight of the tumours in the former group were 203 mm^3^ (95% CI: 145-298 mm^3^) and 272 mg, respectively, whereas tumours in the latter group were 632 mm^3^ (95% CI: 451-962 mm^3^) and 874 mg (Figure 4A and 4B). Immunohistochemistry staining demonstrated that the expression level of p-Src and Src in the treated mice was drastically lower compared with the untreated mice (Figure 4C). Taken together, our data indicated that rhodomycin A influences not only the physical properties of the tumours but also the biochemistry. To investigate the effect of rhodomycin A combined with gefitinib, the gefitinib-resistant lung adenocarcinoma cell lines A549, PC9/gef, and H1975 were treated with different combinations of concentrations of gefitinib and rhodomycin A for 72 hours, and the data were subjected to CI-isobologram analysis. The results showed a synergistic interaction between rhodomycin A and gefitinib in the A549 (CI: 0.052~0.785), PC9/gef (CI: 0.057~0.708), and H1975 cell lines (CI: 0.349~0.824) (Supplementary Table S2–S4). In the A549 and PC9/gef cells, 0.01, 0.05, and 0.1 μM rhodomycin A combined with low-dose gefitinib (0.01 or 0.05 μM) had a synergistic effect. Moreover, the concentration of 0.05 or 0.1 μM rhodomycin A used in the combination treatment rendered the H1975 cells more sensitive to gefitinib, even down to 1 μM. Overall, the results revealed that 0.05 μM and 0.1 μM rhodomycin A can synergistically sensitise A549, PC9/gef, and H1975 lung cancer cells to a wide range of gefitinib treatment concentrations (Figure 4D).

### Effect of rhodomycin A on Src downstream pathways

**Figure 5:**
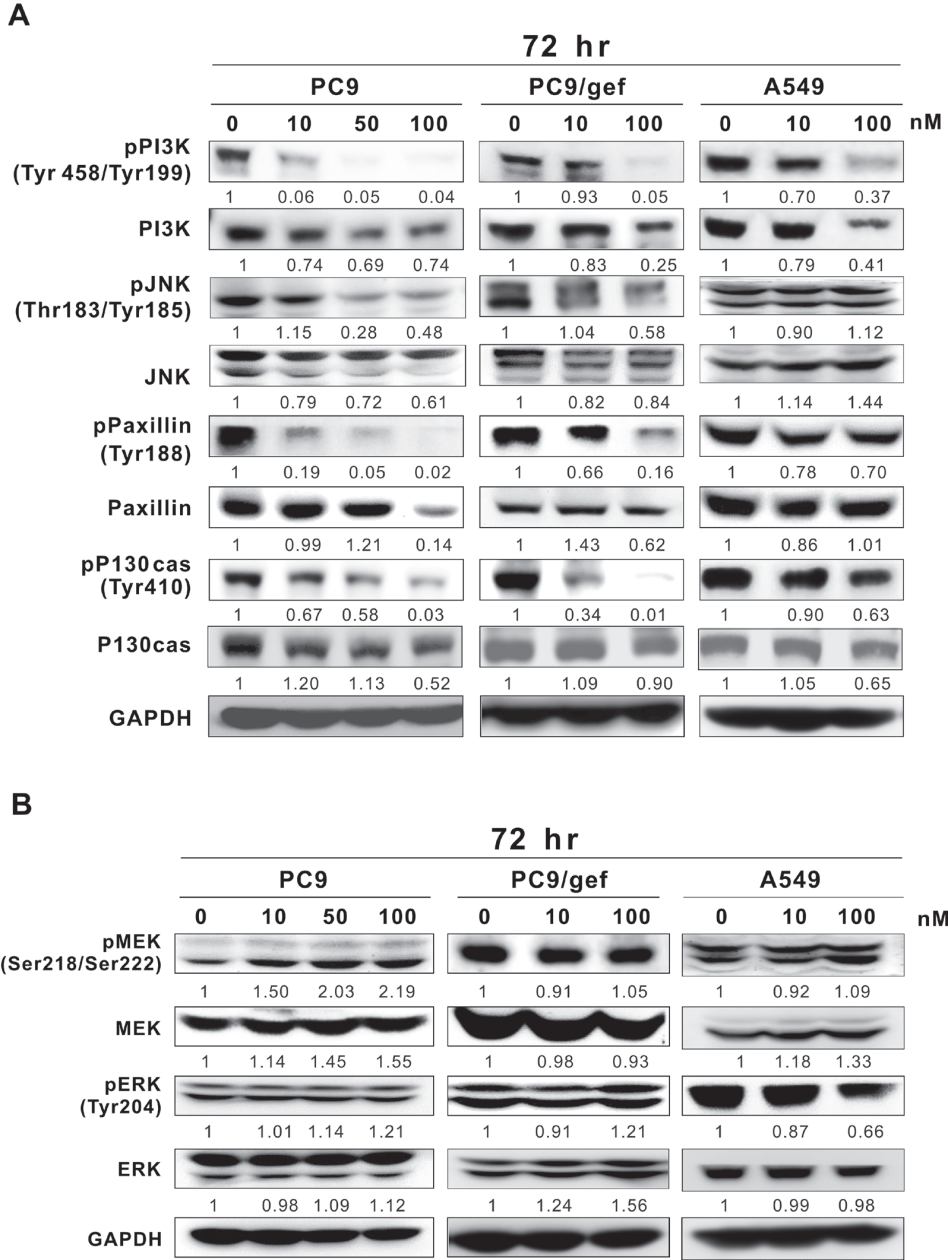
Western blot analyses of Src-downstream proteins in lung cancer cell lines after rhodomycin A treatment The cells were treated with the designated concentrations of rhodomycin A for 72 hours and subjected to Western blot analysis with the indicated antibodies. GAPDH was used as a loading control. **A.** The phosphorylation and protein expression levels of PI3K, JNK, Paxillin, and p130cas in PC9, PC9/gef, and A549 cells. **B.** The phosphorylation and protein expression levels of MEK and ERK in various cell lines. Each treatment was independently performed in triplicate; 0 nM indicates 0.1% DMSO.

Because Src influences many downstream proteins, such as STAT3, PI3K, JNK, Paxillin, p130cas, MEK, and ERK [18], we further investigated whether rhodomycin A impacts any of these proteins. After exposing gefitinib-sensitive PC9 cells to rhodomycin A, we detected both diminished phosphorylation of PI3K, JNK, Paxillin, and p130cas and decreased protein expression in a dose-dependent manner (Figure 5A). A similar pattern was also observed in gefitinib-resistant PC9/gef cells. However, in the EGFR wild-type and gefitinib-resistant A549 cells, only PI3K and p-PI3K were significantly decreased by rhodomycin A treatment. Moreover, no significant changes were detected in the phosphorylation or quantity of MEK and ERK compared with the other Src downstream proteins in these three cell lines (Figure 5B).

### Functional roles of rhodomycin A in protein degradation and transcriptional reduction

**Figure 6:**
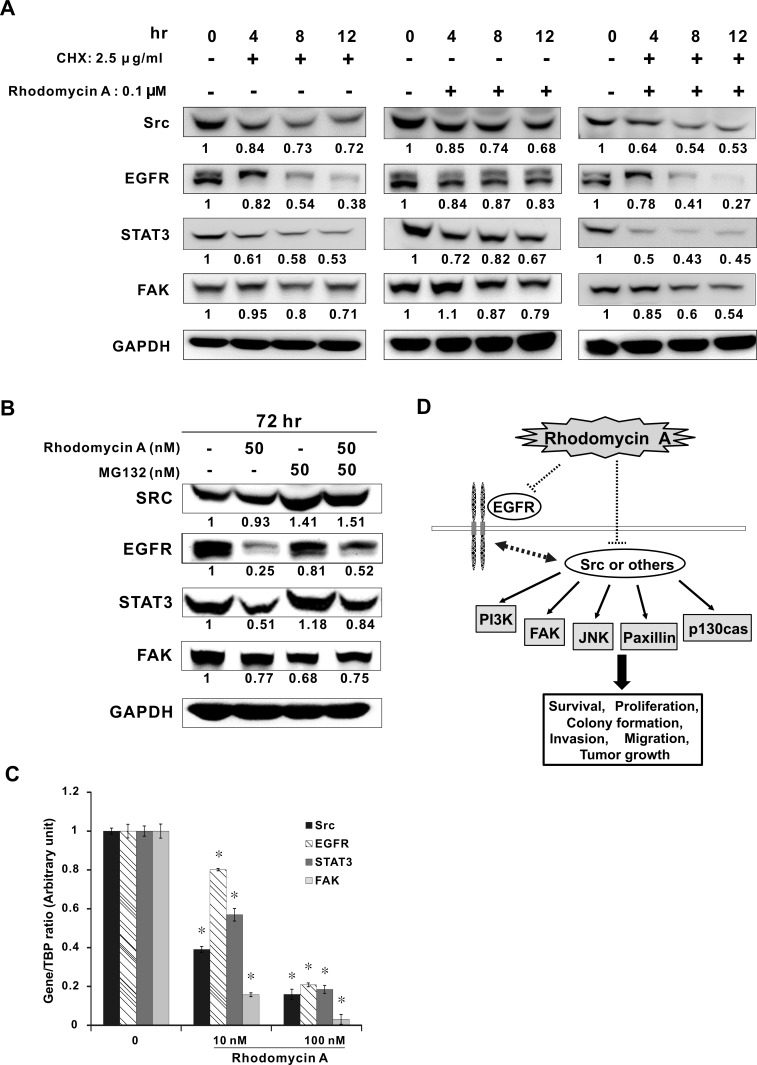
Effects of rhodomycin A on the mRNA and protein levels of Src and its associated proteins **A.** Enhancement of protein degradation by rhodomycin A. Lung cancer PC9 cells were treated with or without the protein synthesis inhibitor cycloheximide (CHX) and/or rhodomycin A for the designated time and then subjected to Western blot analysis for Src, EGFR, STAT3, and FAK. **B.** Promotion of ubiquitination by rhodomycin A. PC9 cells were treated with or without the *proteasome inhibitor* MG132 and/or rhodomycin A for 72 hours and then subjected to Western blot analysis. GAPDH was used as a loading control. **C.** Transcriptional inhibition of rhodomycin A. PC9 cells were treated with rhodomycin A for 72 hours, followed by real-time RT-PCR to detect the mRNA levels of Src, EGFR, STAT3, and FAK. The results are numerically presented in 2^−ΔΔCt^. TBP was used as an internal control. **D.** A hypothetical model for the role of rhodomycin A in suppressing lung cancer progression. The dotted lines mean the direct or indirect effect. Each treatment was independently performed in triplicate; 0 nM indicates 0.1% DMSO. *P < 0.05 compared with control (0 nM, 0.1% DMSO).

The above results revealed that the expression levels of Src and its related proteins were reduced, which suggested that rhodomycin A may trigger protein degradation. After treatment with CHX, the Western blotting results showed that the protein levels of Src, EGFR, STAT3, and FAK are decreased (Figure 6A, left panel). Similar trends were also observed after adding rhodomycin A (Figure 6A, middle panel). Importantly, the simultaneous treatment of CHX and rhodomycin A significantly augmented the degradation of the tested proteins (Figure 6A, right panel). These data implied that rhodomycin A could promote protein degradation.

To examine whether rhodomycin A facilitated protein degradation via the ubiquitin-proteasome system, we used MG132, an inhibitor targeting the 26S proteasome, to interrupt this pathway. Our data showed that the expression levels of Src, EGFR, and STAT3 but not FAK were at least partially restored in the cells treated with rhodomycin A and MG132, in contrast to those treated with rhodomycin A alone (Figure 6B). To explore the effect of rhodomycin A on the transcription level of the tested genes, their expression levels were evaluated with real-time PCR. The results demonstrated that rhodomycin A could significantly reduce the mRNA expression of Src, EGFR, STAT3, and FAK, even at a relatively low concentration (Figure 6C).

## DISCUSSION

The aberrant activation of Src, a typical non-receptor tyrosine kinase (nRTK), has been reported in many cancers, including lung cancer, prostate cancer, pancreatic cancer, breast cancer, and colorectal cancer [19]. A previous study showed that this event contributes to higher drug resistance in patients with lung adenocarcinoma cells [20]. Src overexpression is observed in 50-80% of NSCLC patients and is related to poor prognosis. Therefore, Src can be used as a target to treat lung cancer [21].

Computer-aided drug design, such as pharmacophore modelling and molecular docking, is a powerful tool to evaluate the interactions between drugs and targets and accelerate drug screening [22]. In this study, we identified rhodomycin A as a promising candidate compound for inhibiting Src activity and NSCLC from the NCI compound library using a molecular docking strategy. Further investigations showed that rhodomycin A significantly inhibits *in vitro* cellular functions and *in vivo* tumourigenicity of NSCLC cells, as well as exerts a synergistic effect on tumour growth. Little is known about rhodomycin A because there are few publications on this compound. This chemical is a member of the anthracycline family, which has antitumour activities [23]. Anthracyclines originated from chemotherapy agents extracted from *Streptomyces peucetius* [24]. Among them, doxorubicin is the most well-known, and it has exhibited better performance in cancer treatment [25]. However, the antitumour action of rhodomycin A remains unclear. Our data revealed its multi-functional role and possible involvement in signalling pathways. To the best of our knowledge, this is the first report suggesting that rhodomycin A suppresses NSCLC malignancy through modulating multiple Src-related signalling pathways.

EGFR overexpression is detected in 40-80% of NSCLC. EGFR is essential for regulating cell proliferation, shedding light on lung cancer treatment [26]. In NSCLC, mutations on the EGFR kinase domain constitutively activate EGFR and its downstream signalling pathways, making cells lose control over proliferation [27]. Because there are interactions between Src and EGFR, the suppression of Src may interrupt the downstream signalling pathways of EGFR, such as inducing apoptosis in EGFR mutation cell lines [28]. Additionally, suppressing Src increases the expression level of E-cadherin, improving the effectiveness of EGFR-TKIs [29].

Many Src inhibitors have been created for cancer treatment [16]. Among them, dasatinib has been used to treat patients with chronic myeloid leukaemia [30] and can improve the antitumour capacity of cisplatin in NSCLC cell lines [10]. Nonetheless, the effectiveness of dasatinib is poor in both lung cancer A549 cells with wild-type EGFR or in H1975 cells harbouring L858R and T790M mutations [28], which is similar to the medical outcome of gefitinib treatment. Interestingly, rhodomycin A has activity in all lung cancer cell lines (A549, PC9/gef, and H1975) without particular selectivity for the EGFR status in cytotoxicity. Moreover, it had a higher IC_50_ in the non-tumourigenic human bronchial epithelial cells (BEAS2B) than the tumour cell lines used in this study.

In the murine xenograft model, several mice died after the 4^th^ drug dose, although rhodomycin A could reduce tumourigenicity, Src activity, and Src expression. We speculated that this effect may be due to its metabolites or accumulated toxicity. A previous report indicated that not only the dosage but also the duration of taking anthracyclines increases the chances of heart failure [31]. Therefore, the deaths of those mice were likely associated with heart failure. Because this concern is beyond the scope of our research, we did not investigate this issue further.

The dual inhibition of Src and EGFR activity is a reasonable concept that may benefit NSCLC patients with acquired EGFR resistance mutation. A previous paper demonstrated that doxorubicin, an anthracycline glycoside derivative, can synergise with gefitinib and result in enhanced antitumour activity against the adrenal neuroblastoma of transgenic mice [32]. Unfortunately, a phase II clinical trial of dasatinib combining erlotinib or gefitinib for lung adenocarcinoma patients with acquired resistance mutation did not have positive results [33]. Our findings perhaps provide a potential candidate compound for the replacement of dasatinib in the combination therapy of a Src inhibitor and EGFR-TKI. Our *in vitro* data indicated that rhodomycin A could substantially sensitise gefitinib-resistant lung adenocarcinoma cells (A549, PC9/gef, and H1975) to gefitinib treatment, implying a potential benefit for the clinical application of this compound in reducing the dose of gefitinib. The synergistic effect of TKI treatment combined with rhodomycin A may decrease the cost of targeted therapy drug and patient load.

Participating in many signalling pathways, Src plays an important role in promoting tumour growth, and elevating the tumours' capacity for proliferation, angiogenesis, invasion, migration, and metastasis [17]. Our study demonstrated that rhodomycin A inhibits cellular functions and prevents tumour growth. On signal transduction, Src influences the activities of PI3K, STAT3, FAK, JNK, Paxillin, p130cas, MEK, and ERK, which are widely considered to be essential for cell growth, angiogenesis, and migration [18]. In cancer cell survival, the previous studies showed that RTK and Src mediate cell survival and regulate cell cycle progression through activating the PI3K/AKT pathway [34]. Moreover, the PI3K signalling cascade is involved in a broad range of cancer-related cellular processes [35]. In our study, rhodomycin A not only inhibited Src and EGFR activity but also suppressed PI3K phosphorylation and expression in EGFR mutant (PC9 and PC9/gef) and wild-type (A549) cell lines. In cancer cell migration, several reports have indicated that the FAK-Src complex promotes activities of many FAK-associated Src substrates, including p190RhoGAP, paxillin, and p130cas, which play an important role in the reorganisation of the actin cytoskeleton and motility [36]. Furthermore, Jun N-terminal kinase (JNK) activation would lead to the transcriptional activation of MMP-2 and MMP-9, favouring proteolysis and invasion [37]. Our data revealed that rhodomycin A inhibits FAK, JNK, Paxillin, and p130cas activity and the protein expression in PC9 and PC9/gef cell lines, which may cause the decrease of cancer cell invasion and migration ability. In cancer cell growth, MEK and ERK may be involved in the pathway activated by RTKs and integrins and further stimulate mitogenesis [38]; however, no changes were detected in either MEK and ERK protein expression or phosphorylation in the tested cell lines, except for in A549 cells at a higher treatment dose. Additionally, rhodomycin A downregulated RNA expression and promoted protein degradation, which may have occurred via the ubiquitin-proteasome pathway, which is a common feature for antitumour drugs [39]. Further investigations are needed to determine the detailed mechanisms.

Although we suggest that rhodomycin A can affect Src and subsequently, downstream-related proteins through Src inhibition, we cannot rule out the possibility that rhodomycin A influences multiple targets. Previous reports have indicated that multi-target drugs compensate for the disadvantages of single target counterparts in disease treatment. For example, imatinib and sunitinib, which are used on gastrointestinal stromal tumours (GISTs), simultaneously interrupt BCR-ABL, KIT, and PDGFR tyrosine kinase pathways, promoting cell cycle arrest [40, 41]; sorafenib inhibits the VEGFR, PDGFR, KIT, FLT3, and RAF pathways in late-stage kidney cancer [42]. In this study, rhodomycin A inhibited cancer cell proliferation, clonogenicity, motility, and invasiveness, showing that it may possess a multi-functional effect, which could contribute to its benefits as a cancer treatment. Therefore, as a single- or multi-target drug, rhodomycin A may be useful in the development of future therapeutic drugs, such as a lead compound.

## MATERIALS AND METHODS

### Cell culture and drug treatment

The human bronchial epithelial cell line BEAS2B (ATCC CRL-9609) and human lung cancer cell lines A549 (ATCC CCL-185) and H1975 (ATCC CRL-5908) were purchased from the American Type Culture Collection (ATCC, Manassas, VA, USA); human lung cancer cell lines PC9 and PC9/gef were kindly provided by Dr. Chih-Hsin Yang at the NTU Hospital. These cell lines were maintained at 37°C in a humidified atmosphere of 5% CO_2_ using RPMI-1640 media (Gibco, Carlsbad, CA) supplemented with 10% foetal bovine serum (FBS; Gibco) and 1% penicillin/streptomycin (Gibco). Rhodomycin A (NSC-136044) was acquired from the National Cancer Institute (NCI, Bethesda, MD, USA) and preserved at −20°C in DMSO for a final concentration of 0.1 M.

### Real-time PCR analysis

The mRNA expression levels of Src and related genes were detected on an ABI prism 7300 sequence detection system (Applied Biosystems, Calsbad, CA, USA) using the SYBR Green approach (Roche, Nutley, USA). Briefly, the PCR reagent, primers, and cDNA template were gently mixed and then subjected to PCR reaction. TATA-box binding protein (TBP) was used as the internal control (GenBank X54993). The detailed procedures and calculations have been previously described [43].

### Western blotting

Western blot analysis was used to determine the protein phosphorylation and expression after rhodomycin A treatment. The detailed procedures were as previously described [44]. Briefly, the membrane was incubated with primary antibody at a different dilution in antibody diluent buffer overnight at 4°C. Anti-GAPDH (Upstate Biotechnology, Lake Placid, NY, USA) was used as a loading control. EGFR, STAT3 (F-2), PI3K, phospho-MEK1/2 (Ser218/Ser222), MEK, phospho-ERK (Tyr204), ERK, Paxillin, and p130cas were purchased from Santa Cruz Biotechnology, Inc. (Dallas, TX, USA); phospho-Src (pY418), phospho-FAK (Tyr576), and FAK were purchased from Invitrogen (Carlsbad, CA, USA); phospho-EGFR (Tyr1068), phospho- STAT3 (Tyr705), phospho-PI3K (Tyr458), phospho-SAPK/JNK (Thr183/Tyr185), SAPK/JNK, phospho-Paxillin (Tyr118), and phospho-p130cas (Tyr410) were purchased from Cell Signaling Technology (Danvers, MA, USA); and the primary antibody for Src was made in house (ATCC CRL-2651). The membrane was washed 3 times with wash buffer and incubated in HRP-conjugated IgG antibody (diluted 1:5000) at room temperature. The reaction was stopped by washing the membrane three times with PBST and then photographed with the UVP AutoChemi Image System (UVP, Upland, CA, USA). Where appropriate, the Western blot data were quantified by image system and normalized with the loading control. The relative fold changes were shown below the blots.

### Cell viability assay and proliferation assays

The PrestoBlue^®^ (Invitrogen) cell viability reagent was used to determine the cytotoxic impact of the compound or cell proliferation ability according to the manufacturer's protocol. Cells were seeded onto a 96-well plate (2.5×10^3^/100 μl) and cultured at 37°C for 12-16 hours. After removing the cultured media, 100 μl of drugs with the desired concentration was added to each well. After 24, 48, and 72 hours, 10 μl of PrestoBlue reagent was added, and the cells were allowed to rest for 1 hour at 37°C before absorbance at 570/600 nm measured using an ELISA Reader (Vector^3^; Perkin-Elmer, Santa Clara, CA, USA).

### Colony formation

To determine the clonogenicity of cancer cells, anchorage-dependent and -independent approaches were used as described previously [45]. For the anchorage-independent growth assay, 2 ml of 0.7% LMP agarose was poured onto a plate to create a basal layer. As the basal layer solidified, 100 μl of 1×10^4^/ml cells were seeded with 900 μl of culture media and 1 ml of 0.7% LMP agarose. After a gentle mixing, drugs of the desired concentration were added. Once the colonies formed, 0.5 mg/ml of p-iodonitrotetrazolium violet was added to stain the colonies. For the anchorage-dependent growth assay, 500 cells were seeded in a culture dish that had culture media and drug solution. When the colonies formed after 7-10 days, the cells were washed using 1xPBS and fixed for 15 minutes using methanol. The 0.005% crystal violet was added to stain the colonies for > 8 hours. After the colonies were photographed, the number of colonies with a diameter larger than 0.1 mm was calculated.

### Migration and invasion assay

A transwell membrane (8 μm pore size, 6.5 mm diameter; Corning Costar Corporation, MA) coated with or without Matrigel (2.5 mg/ml; BD Biosciences, San Jose, CA) was used for invasion and transwell migration assays as described previously [46]. The upper wells were filled with serum-free medium and cells (2×10^4^ or 5×10^3^ cells per well). The lower wells of the transwells contained the same medium supplemented with 10% FBS.

### Tumourigenesis and immunohistochemistry assay

Sixteen four-week-old severe combined immunodeficiency (SCID) nude mice were purchased from the National Laboratory Animal Center (NLAC, Taipei, Taiwan). We achieved tumour growth in the mice according to previously described protocols [47]. In total, 4×10^6^ live PC9/gef cells were injected subcutaneously into the nude mice. Tumor volume was assessed weekly until volumes reached an average of 100 mm3. To examine the effects of drugs on tumour suppression, the mice were grouped into DMSO-treated (with 0.1% DMSO) and drug-treated (with 0.25 mg/kg of rhodomycin A) groups. Every two days, the former was injected with 100 μl of PBS with 0.1% DMSO, whereas the latter was injected with drugs. After 6-7 weeks, the mice were sacrificed using CO_2_, and their tumour volumes were estimated from their caliper-measured lengths (a) and widths (b) using the formula V = 0.4 × ab^2^ [48]. The mouse experiments were approved by the Institutional Animal Care and Use Committee of the National Chung Hsing University. Immunohistochemistry analysis was performed on the paraffin-embedded tumor tissue samples using p-Src and Src staining. Briefly, rabbit anti-human p-Src polyclonal antibody (Invirogen, Carlsbad, CA, USA) and anti-human Src monoclonal antibody (Abcam, Cambridge, UK) were used in the primary reaction. The DAKO EnVision System, containing a secondary horseradish peroxidase-conjugated anti-rabbit antibody complex, was used with 3,3′-diaminobenzidine to detect the p-Src and Src.

### Protein degradation experiments

Cells (3.5×10^5^) were incubated overnight in a culture dish; then, the cells were treated with 0.1 μM rhodomycin A and/or 2.5 μg/ml cycloheximide (CHX) (Sigma-Aldrich, St. Louis, MO, USA), an inhibitor to stop protein synthesis. After 4, 8, and 12 hours, the cell lysates were extracted and subjected to Western blot analysis of the tested proteins. Furthermore, to investigate whether the protein degradation occurs through ubiquitination, the cells were harvested after incubation with 50 nM MG132 (Sigma-Aldrich), a proteasome inhibitor, for 24 hours or 50 nM rhodomycin A for 72 hours.

### Drug synergy analysis

To determine the combined effect of various concentrations of rhodomycin A and gefitinib on A549, PC9/gef and H1975 cytotoxicity, the data from the proliferation assays were entered into CalcuSyn software (Biosoft, Cambridge, UK), and the combination index (CI)-isobologram method was used as described previously [49]. CI < 1, CI = 1 or CI > 1 represent the synergism, additive effect or antagonism of both compounds, respectively.

### Statistical analysis

The results are presented as the mean ± standard deviation, and all experiments were performed at least in triplicate. All data were analysed for significant differences using either a *T*-test or ANOVA (Excel; Microsoft). *P* values < 0.05 were considered statistically significant.

## SUPPLEMENTARY MATERIAL TABLES AND FIGURES


