# Supplementary material for: Rhodomycin A, a novel Src-targeted compound, can suppress lung cancer cell progression via modulating Src-related pathways
Source: Oncotarget. 2015 Jul 3;6(28):26252–65. doi: 10.18632/oncotarget.4761 (PMC4694899; doi:10.18632/oncotarget.4761)
Supplement: Supplementary file 1 [file oncotarget-06-26252-s001.pdf]

Rhodomycin A, a novel Src-targeted compound, can suppress lung cancer cell progression via modulating Src-related pathways

Supplementary Material

Supplementary Table S1. The top 8 ligands as evaluated by the Libdock score and consensus score derived from molecular docking analysis. The well-known Src inhibitors dasatinib and imatinib were used as references for the selections.

Code name	Compound name (NSC ID)	Consensus score	Libdock score	Binding energy
Selected compounds				
N1	5548	9	162.042	-248.14695
N2	627875	5	152.644	-216.81675
N3	136044	9	163.188	-249.48529
N4	617998	8	167.744	-337.82613
N5	605471	7	153.376	-232.87025
N6	630367	6	157.306	-283.00974
N7	407321	4	148.256	-212.91917
N8	372585	7	151.348	-210.02225
Reference compounds				
	Imatinib	5	126.731	-138.45711
	Dasatinib	3	118.254	-169.02678

Supplementary Table S2. Combination index (CI) and fractional effect (FE) of combination treatment of rhodomycin and gefitinib (Iressa) in lung cancer A549 cells.

**Gefitinibe (μM)**	**Rhodomycin (μM)**					
	**0.01**		**0.05**		**0.1**	
	FE	CI	FE	CI	FE	CI
**0.01**	0.243±0.169	0.197±0.144	0.640±0.028	0.210±0.017	0.935±0.013	0.103±0.015
**0.05**	0.310±0.020	0.137±0.013	0.758±0.011	0.144±0.006	0.949±0.009	0.087±0.011
**0.1**	0.332±0.036	0.150±0.028	0.758±0.011	0.145±0.006	0.976±0.011	0.052±0.016
**1**	0.340±0.060	0.596±0.289	0.811±0.042	0.122±0.025	0.973±0.011	0.055±0.016
**5**	0.468±0.003	0.785±0.018	0.839±0.019	0.118±0.015	0.974±0.007	0.055±0.011

Supplementary Table S3. Combination index (CI) and fractional effect (FE) of combination treatment of rhodomycin and gefitinib (Iressa) in lung cancer PC9/gef cells.

**Gefitinibe (μM)**	**Rhodomycin (μM)**					
	**0.01**		**0.05**		**0.1**	
	FE	CI	FE	CI	FE	CI
**0.01**	0.027±0.012	2.600±0.401	0.312±0.003	0.505±0.008	0.836±0.001	0.089±0.001
**0.05**	0.306±0.024	0.144±0.027	0.425±0.041	0.315±0.055	0.832±0.008	0.091±0.005
**0.1**	0.382±0.040	0.103±0.028	0.396±0.038	0.370±0.066	0.796±0.074	0.118±0.053
**1**	0.352±0.040	0.530±0.256	0.385±0.075	0.708±0.412	0.889±0.037	0.057±0.021
**5**	0.438±0.018	0.702±0.156	0.523±0.011	0.416±0.039	0.880±0.036	0.062±0.021

Supplementary Table S4. Combination index (CI) and fractional effect (FE) of combination treatment of rhodomycin and gefitinib (Iressa) in lung cancer H1975 cells.

**Gefitinibe (μM)**	**Rhodomycin (μM)**					
	**0.01**		**0.05**		**0.1**	
	FE	CI	FE	CI	FE	CI
**1**	0.075±0.014	2.908±0.670	0.352±0.055	1.044±0.307	0.692±0.092	0.352±0.180
**5**	0.263±0.010	1.230±0.071	0.516±0.049	0.659±0.154	0.707±0.001	0.389±0.002
**10**	0.319±0.061	1.632±0.535	0.543±0.035	0.808±0.134	0.762±0.022	0.349±0.050
**25**	0.624±0.029	0.824±0.120	0.774±0.006	0.425±0.018	0.804±0.012	0.419±0.038

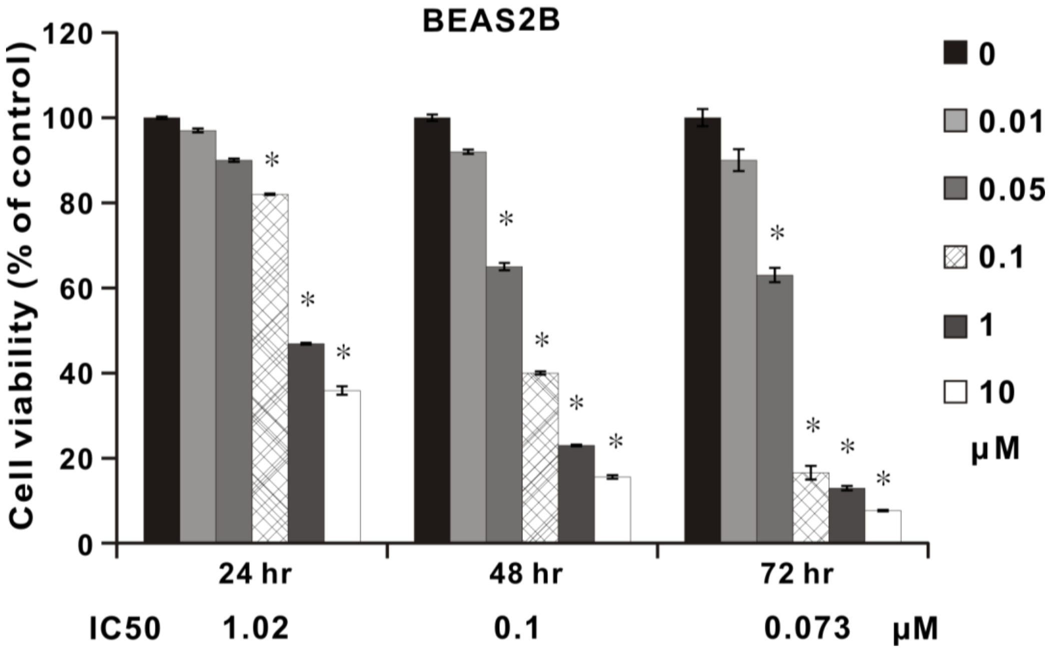

Supplementary Fig S1. The cytotoxic effect of rhodomycin A on non-tumour BEAS2B cells. The cytotoxicity of rhodomycin A on BEAS2B cells was determined by the PrestoBlue® cell viability assay at 24, 48, or 72 hours. The IC_{50} at 24, 48, and 72 hours was 1.02, 0.1, and 0.073 μM, respectively. Each treatment was independently performed in triplicate; 0 μM indicates 0.1% DMSO. *P<0.05 compared with control (0 μM, 0.1% DMSO).

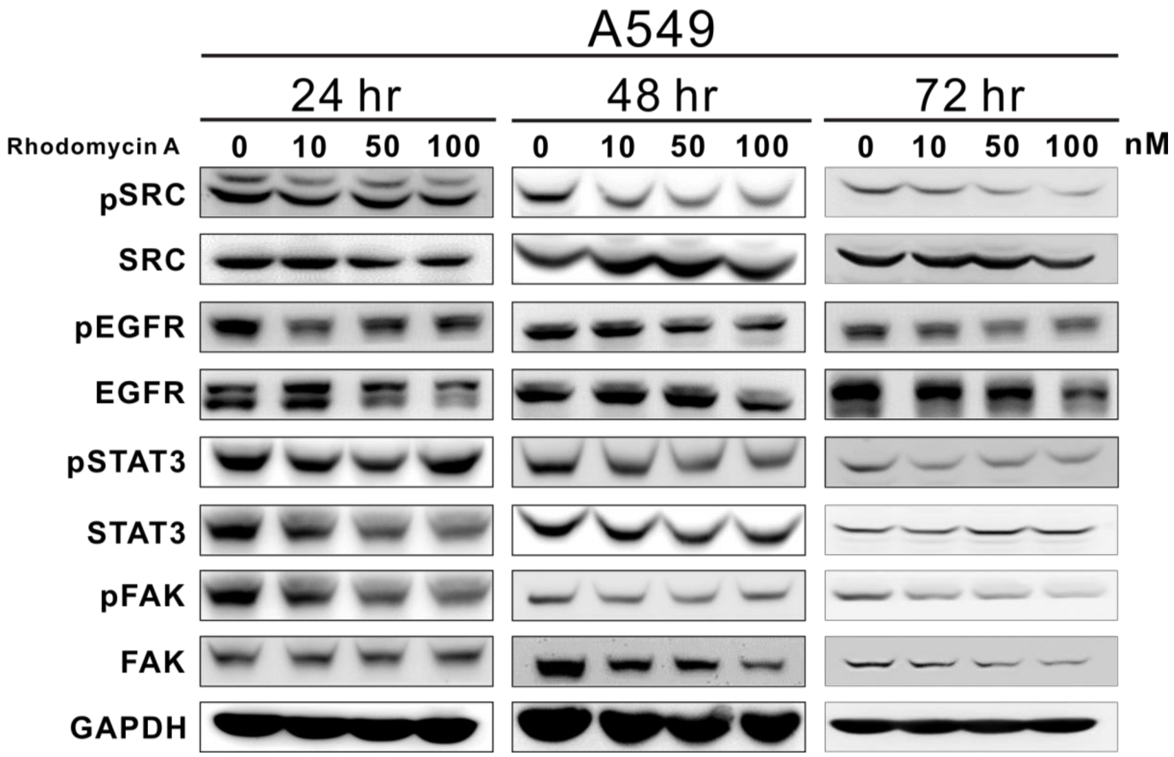

Supplementary Fig S2. Effect of rhodomycin A on Src and its associated proteins in the A549 cell line. A549 cells were treated with rhodomycin A and subjected to Western blot analyses to detect the phosphorylation and expression levels of Src, EGFR, STAT3, and FAK. GAPDH was used as an internal control. Each treatment was independently performed in triplicate; 0 nM represented 0.1% DMSO.

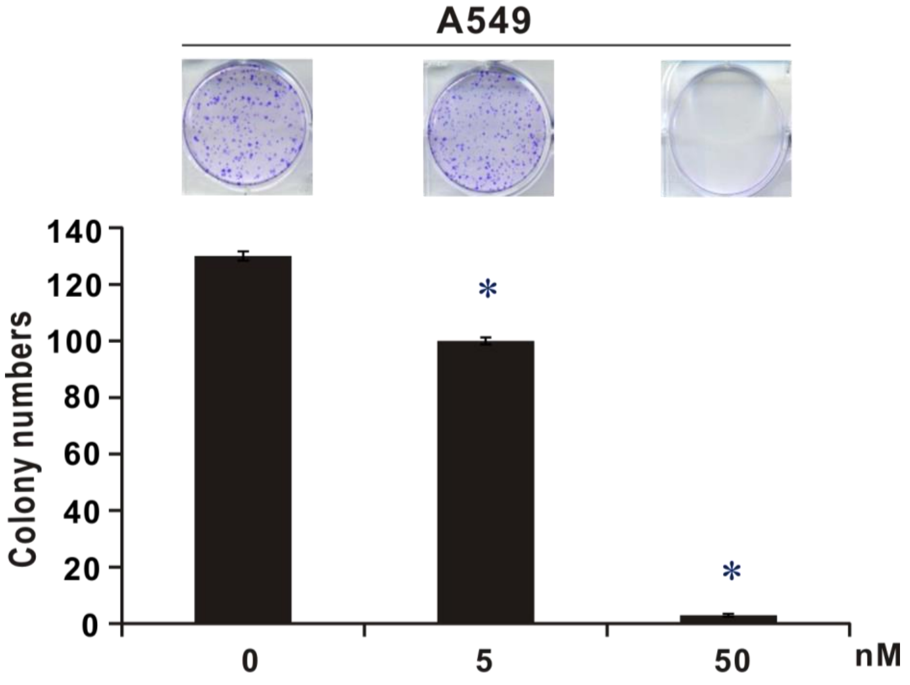

Supplementary Fig S3. Inhibition of anchorage-dependent growth in A549 cells by rhodomycin A. The cells grown in a culture dish without soft agar were treated with the designated concentrations of rhodomycin A. The colonies with a diameter ≥0.3 mm were selected. Each treatment was independently performed in triplicate; 0 nM indicates 0.1% DMSO. *P<0.05 compared with vehicle-treated control (0 nM, 0.1% DMSO).

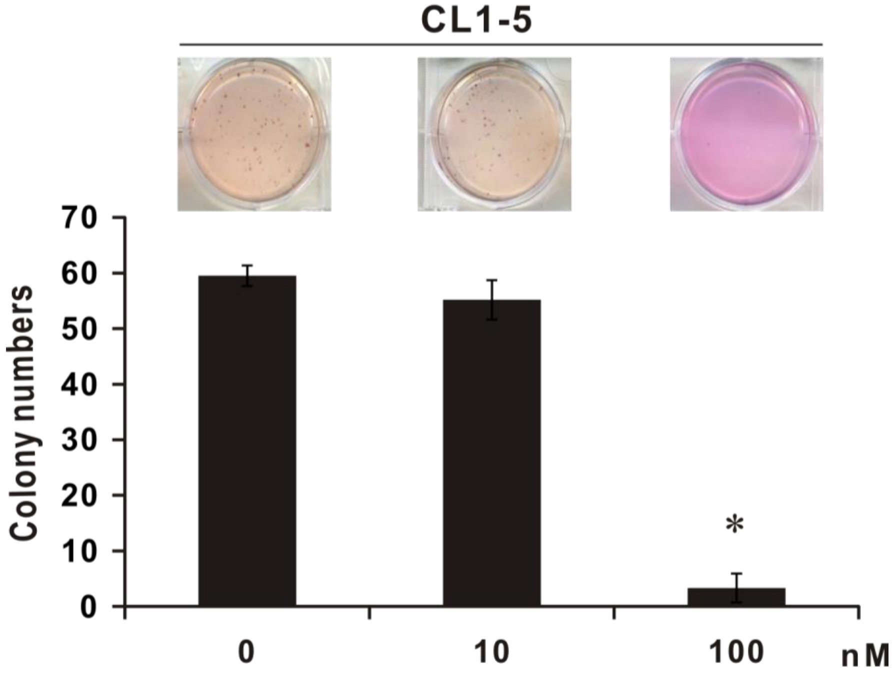

Supplementary Fig S4. Effect of rhodomycin A on anchorage-independent growth of CL1-5 cells. The cells grown in soft agar were treated with rhodomycin A and then evaluated in clonogenic assays. The colonies with diameters ≥0.5 mm were counted. Each treatment was independently performed in triplicate; 0 nM indicates 0.1% DMSO. *$P<0.05$ compared with vehicle-treated control (0 nM, 0.1% DMSO).
